# Effects of the non-native *Arapaima gigas* on native fish species in Amazonian oxbow lakes (Bolivia)

**DOI:** 10.1371/journal.pone.0314359

**Published:** 2025-01-02

**Authors:** Danny Rejas, Monika Winder, Reinaldo Cholima, Thierry Oberdorff

**Affiliations:** 1 Department of Ecology, Environment and Plant Sciences, Stockholm University, Stockholm, Sweden; 2 Unidad de Limnología y Recursos Acuáticos, Universidad Mayor de San Simón, Cochabamba, Bolivia; 3 Centro de Investigación de Recursos Acuáticos, Universidad Autónoma del Beni, José Ballivián, Beni, Bolivia; 4 UMR CRBE (Center for Research on Biodiversity and Environment), CNRS5300, IRD, INP, UPS, Université Paul Sabatier, Toulouse, France; National Museums of Kenya, KENYA

## Abstract

The introduction of non-native fish species into new environments has raised global concerns due to potential ecological impacts on recipient ecosystems. A previous study focusing on the introduced fish species *Arapaima gigas* in Bolivian Amazon waters showed that its isotopic niche significantly overlapped with most co-occurring native fish species, suggesting potential competition. To evaluate this hypothesis, we extended here the investigation by comparing the trophic position and isotopic niche width of eleven abundant native fish species inhabiting both colonized and non-colonized floodplain lakes. We found lower trophic positions in colonized versus non-colonized lakes only for native piscivores, mostly driven by a shift towards increased dietary proportion of detritivorous fishes. Conversely, results showed that the isotopic niche width of most fish species analyzed (i.e. 10 over 11 species) did not significantly decrease in colonized compared to non-colonized lakes. Our overall results suggest potentially low competitive interactions between *A*. *gigas* and native fishes, with the notable exception of piscivorous species. We attribute our findings to the high abundance of available resources in Amazon oxbow lakes.

## Introduction

Introduction of non-native fish species into new environments has become a growing global concern due to their potential ecological effects on recipient ecosystems (see [1] for a review). While these effects may often be subtle, many introductions also exert significant impacts that may range from food webs re-structuring to extirpation of local faunas [2]. *Arapaima gigas*, one of the largest fish in the Amazon Basin, is a prime example of a species introduced outside its natural range primarily for aquaculture, spurred by its significant economic value [3,4]. Originally confined to the floodplains of the Solimões-Amazon, Tocantins-Araguaia and Essequibo Rivers [5,6], *A*. *gigas* has established populations outside its natural distribution range in Brazil, Perú and Bolivia, where it is claimed to be invasive threatening native fish populations and local ecosystems [3].

The Colonization of Bolivian waters by *A*. *gigas* originated in the upper Madre de Dios River in Peru, following its introduction on different occasions in several lakes between the mid-1960s and early 1980s with subsequent colonization processes towards the Bolivian territory during flood events. Multiple secondary introductions from aquaculture reinforced this process [7–10]. At present, *A*. *gigas* has been reported virtually in all the Bolivian tributaries of the Madre de Dios and Orthon Rivers and in the lower reaches of the Beni, Mamoré and Iténez Rivers [10]. The expansion of the colonization into the Mamoré River basin appears to have been dampened by rapids near Guayaramerín. Nonetheless, the colonization of the upper reaches of Mamoré River seems imminent, fueled by unregulated trade and the proliferation of aquaculture activities throughout the region [11].

Although *A*. *gigas* is often considered an apex predator [10,12,13] due to its large body size and piscivorous habits [14,15], recent studies in both colonized [16] and natural habitats [17,18] reveal that it exhibits high trophic plasticity, acting more as an omnivore with piscivorous tendencies. Moreover, it has been shown that the isotopic niches (a proxy for trophic niche [19–21] of *A*. *gigas* and of the native fish species inhabiting two recently colonized oxbow lakes of the Madre de Dios River overlapped substantially, suggesting a potential for competition [16]. Yet, niche overlap in itself does not necessarily result in competition when resources are sufficiently abundant [22,23]. To go a step further, here, we compare the TP and isotopic niche width of eleven most abundant native species of diverse trophic levels in two colonized and one currently non-colonized floodplain lakes of the Bolivian Amazon. Given that the broad isotopic niche of *A*. *gigas* overlaps with the one of most native fish species [16], we hypothesize that *A*. *gigas* presence may *i*) decrease TPs of native fishes by forcing them to feed lower on the food chain (i.e. reducing their δ^15^N-values [24] and/or *ii*) reduce their isotopic niche width [22,25] by shrinking their dietary range (i.e. narrowing their range in δ^13^C-values [26]).

## Materials and methods

ULRA/UMSS is an Authorized Scientific Institution (ICA) accredited by the Bolivian Dirección General de la Biodiversidad y Áreas Protegidas (DGBAP) to conduct biological scientific research within the Bolivian territory (Resolución administrativa BMABCC 026/09). Fishes were manipulated according to procedures permitted by the Viceministerio de Medio Ambiente. This study did not involve species classified as either endangered or protected according to the Bolivian red list.

**Fig 1:**
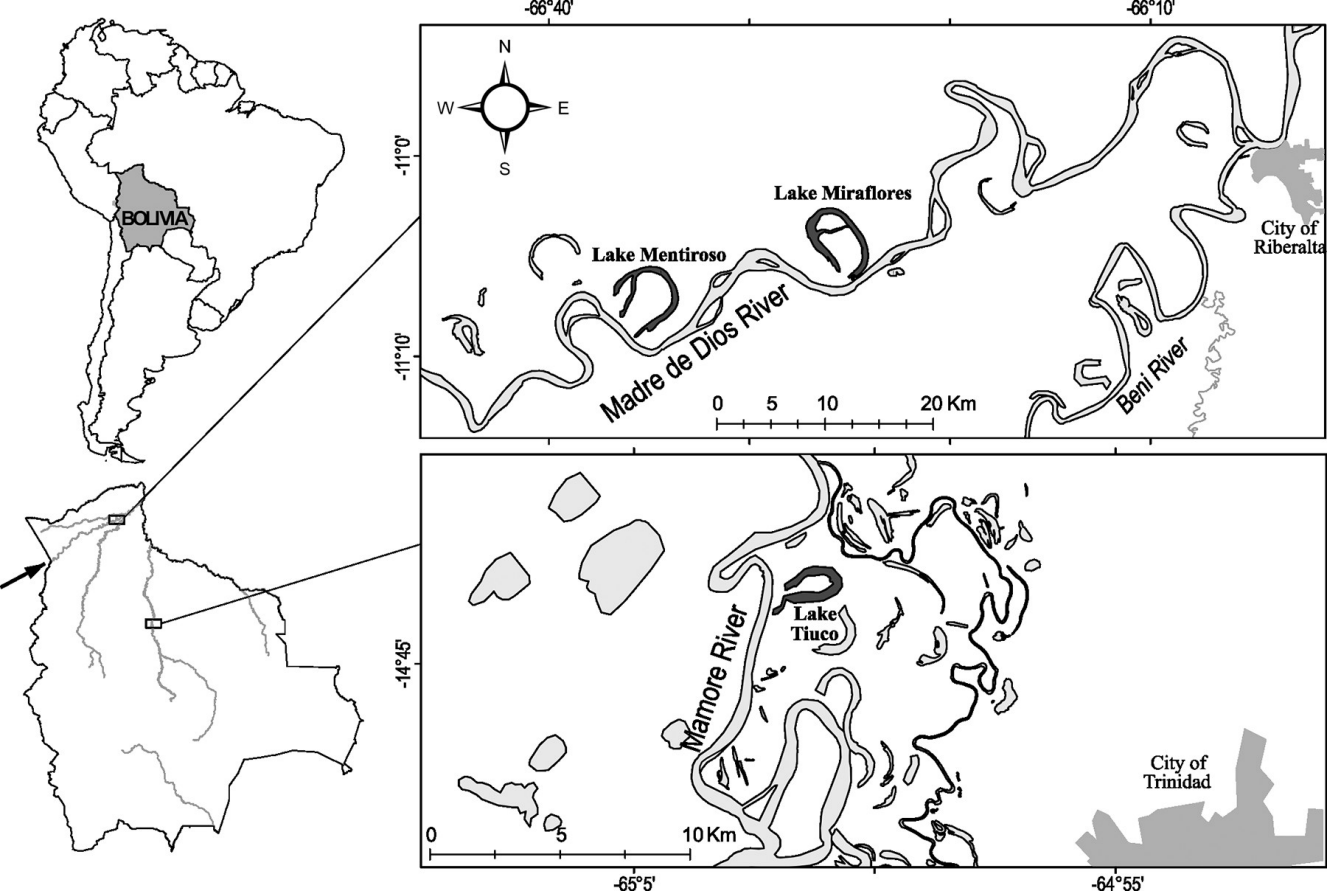
Study area. Location of the lakes studied (black filled) in the floodplain of Madre de Dios and Mamoré rivers in Bolivia, South America. The black arrow shows the direction of colonization of *Arapaima gigas*. Redrawn from OpenStreetMap data (© OpenStreetMap contributors, licensed under ODbL) and partially adapted from Rejas et al. 2023 [16] © 2023 Published by John Wiley & Sons Ltd. under the terms of the Creative Commons Attribution License CC BY 4.0.

We used stable isotope data of fishes inhabiting one oxbow lake located in the floodplain of Mamoré River and where *A*. *gigas* was absent. These stable isotope data were compared with recently published data from two oxbow lakes located in the floodplain of Madre de Dios River [16] where *A*. *gigas* is abundant and represents nearly 50% of the total commercial catches [11]. Samples in the Mamoré River were collected in November 2015 (Lake Tiuco) and samples in the Madre de Dios River were collected in October 2015 (Lake Mentiroso) and July 2017 (Lake Miraflores). Madre de Dios and Mamoré rivers are the main tributaries of the Madera River (Fig 1), both are white-water rivers characterized by turbid, ochre-colored waters with high loads of suspended sediments [27]. White-waters are known to be rich in resources diversity and availability (e.g., nutrients, zooplankton, aquatic insects, fishes) [28], thereby supposedly reducing the likelihood of competitive exclusion [22]. The three sampled lakes were located close to the main channel of the river (< 300 m). The same sampling and stable isotope analyses procedures were applied for the three lakes (described in [16]).

We collected samples during the low-water period, when floodplain lakes are isolated from the river. Fishes were captured by local fishermen, fishing with hook and lines and a set of gillnets with knot-to-knot distances from 30 to 100 mm. To minimize suffering, recommended humane euthanasia methods for fish were applied. Small and medium-sized specimens were immersed in an ice slurry [29], while large fish were euthanized by percussive stunning, performed by trained fishermen [30].

We collected samples of adult individuals only to avoid potential biases due to species ontogenetic dietary shifts [17]. Mean standard lengths are provided in S1 Table. In total, we captured 20 native fish species, 19 in the colonized lakes (Lakes Mentiroso and Miraflores) and 18 in the non-colonized lake (Lake Tiuco), with 17 species common to both types of lakes. We retained 11 fish species from which we captured sufficient individuals to perform analyses in both locations (≥ 4 individuals). Based on available literature data on gut content analyses [31–33] we further classified these 11 species into four trophic guilds: detritivores, herbivores, invertivores and piscivores to test for differences in TP between trophic guilds and between colonized and non-colonized lakes (Table 1).

**Table 1:** Sample size (n) and mean (± SD) of ẟ^13^C, ẟ^15^N, ẟ^13^C_corr,_ and trophic position (TP) values, and minimum and maximum ẟ^13^C and ẟ^15^N values for 11 native fish species from non-colonized (River Mamoré) and colonized (Madre de Dios River) lakes. Species were assigned to trophic guilds based on available literature.

	n	δ^13^C	δ^13^C_min_	δ^13^C_max_	δ^13^C_corr_	δ^15^N	δ^15^N_min_	δ^15^N_max_	TP
	Non-colonized								
Detritivores									
*Potamorhina altamazonica*	8	-34.0 ± 2.3	-38.4	-31.0	-0.2 ± 0.2	7.0 ± 0.9	5.7	8.6	2.2 ± 0.3
*Potamorhina latior*	10	-36.5 ± 1.8	-39.3	-33.5	-0.4 ± 0.1	7.0 ± 0.6	6.1	7.9	2.2 ± 0.2
Herbivores									
*Colossoma macropomum*	9	-31.0 ± 2.3	-35.0	-28.2	0.0 ± 0.2	7.6 ± 1.0	6.5	9.2	2.4 ± 0.4
*Mylossoma duriventre*	13	-27.9 ± 1.3	-29.9	-25.9	0.3 ± 0.1	6.9 ± 1.0	5.1	8.2	2.2 ± 0.4
*Piaractus brachypomus*	10	-30.1 ± 1.8	-32.0	-26.9	0.1 ± 0.1	6.4 ± 0.7	5.2	7.6	2.0 ± 0.3
*Prochilodus nigricans*	8	-31.9 ± 2.6	-35.7	-28.6	0.0 ± 0.2	8.1 ± 0.7	6.7	8.8	2.6 ± 0.3
Invertivores									
*Triportheus albus*	12	-28.3 ± 1.5	-32.0	-26.4	0.2 ± 0.1	8.4 ± 0.6	7.4	9.3	2.7 ± 0.2
Piscivores									
*Hoplias malabaricus*	9	-29.6 ± 1.9	-33.3	-27.6	0.2 ± 0.1	10.5 ± 0.6	9.5	11.4	3.5 ± 0.2
*Pseudoplatystoma fasciatum*	12	-29.0 ± 1.3	-30.8	-26.5	0.2 ± 0.1	10.3 ± 0.7	9.2	11.2	3.4 ± 0.2
*Serrasalmus spilopleura*	10	-27.1 ± 0.9	-27.9	-25.1	0.3 ± 0.1	10.3 ± 0.4	9.6	11.0	3.4 ± 0.2
*Plagiscion squamosissimus*	17	-30.2 ± 1.7	-33.4	-27.8	0.1 ± 0.1	10.8 ± 0.5	9.9	12.0	3.6 ± 0.2
	Colonized								
Detritivores									
*Potamorhina altamazonica*	10	-31.8 ± 0.7	-32.9	-31.0	-0.1 ± 0.1	6.0 ± 0.8	5.0	7.3	2.0 ± 0.3
*Potamorhina latior*	12	-32.0 ± 1.9	-34.3	-28.7	-0.2 ± 0.2	6.0 ± 0.9	4.9	7.5	2.0 ± 0.3
Herbivores									
*Colossoma macropomum*	12	-30.0 ± 1.4	-32.0	-28.2	0.1 ± 0.2	6.7 ± 0.9	5.4	7.8	2.2 ± 0.3
*Mylossoma duriventre*	13	-29.0 ± 1.9	-31.8	-26.0	0.2 ± 0.2	6.8 ± 0.7	5.8	7.9	2.3 ± 0.2
*Piaractus brachypomus*	4	-30.4 ± 0.5	-31.1	-30.0	0.0 ± 0.1	7.2 ± 0.5	6.5	7.9	2.4 ± 0.2
*Prochilodus nigricans*	8	-35.0 ± 1.5	-37.5	-32.9	-0.1 ± 0.1	8.3 ± 0.4	7.8	8.9	2.0 ± 0.1
Invertivores									
*Triportheus albus*	9	-31.3 ± 0.9	-32.7	-30.2	-0.1 ± 0.1	7.6 ± 1.1	5.2	9.4	2.6 ± 0.4
Piscivores									
*Hoplias malabaricus*	5	-31.8 ± 1.6	-33.3	-29.8	-0.1 ± 0.2	9.5 ± 0.5	9.0	10.1	3.2 ± 0.2
*Pseudoplatystoma fasciatum*	9	-33.1 ± 1.3	-34.7	-31.3	-0.3 ± 0.2	9.0 ± 0.5	8.2	9.7	3.0 ± 0.2
*Serrasalmus spilopleura*	6	-31.0 ± 1.7	-33.5	-29.5	-0.1 ± 0.2	8.5 ± 0.5	8.0	9.2	2.9 ± 0.2
*Plagiscion squamosissimus*	17	-33.7 ± 2.0	-38.4	-29.8	0.0 ± 0.2	11.6 ± 0.4	11.0	12.6	3.2 ± 0.2

For stable isotope analyses, a sample of ~10 g of muscle tissue was taken from the dorsal part of each fish individual. All samples were rinsed with deionized water and stored frozen in cryovials. Posteriorly samples were freeze-dried and ground to a fine powder using a mortar and a pestle. Approximately 1 mg of dry sample material was packed into tin capsules. In addition, we sampled basal carbon sources (C3 and C4 aquatic macrophytes, phytoplankton (as particulate organic matter) and terrestrial vegetation. C3 aquatic macrophytes were not present in Lake Miraflores during the sampling period). δ^13^C and δ^15^N measurements were performed at the UC Davis Stable Isotope Facility laboratory (University of California, Davis, USA).

Stable isotope ratios are reported in parts per thousand (‰) relative to international standards: Pee Dee belemnite (PDB) and atmospheric N for carbon and nitrogen, respectively. Isotope ratios are defined as: δX = (*R*_*sample*_*/R*_*standard*_− 1) × 10^3^; where X represents either carbon or nitrogen [34]. When interpreting isotope data, positive δ values indicate a higher proportion of the heavy isotope compared to the standard, while negative δ values indicate a lower proportion [35]. The standard deviations for replicate measurements of standards were ≤0.13‰ and ≤0.10‰ for δ^13^C and δ^1^⁵N, respectively.

We used two-way ANOVA to assess interaction effects of lake and basal carbon source on δ^13^C and δ^15^N values, using each individual sample as replicates. Since isotopic signatures of basal carbon sources showed spatial variations (see Results), we thus corrected δ^15^N and δ^13^C data for these differences before calculating the isotopic niche width. To this end, we calculated standard ellipse areas (SEAs; [36] metrics using trophic position (TP) instead of δ^15^N, and δ^13^C_corr_ instead of δ^13^C [37,38]. TP was calculated using the equation: TP = 2 + (δ^15^N_fish_ - δ^15^N_base_)/ Δ; where 2 is the TP of the organism used to estimate the baseline (a primary consumer) and Δ is the N isotopic fractionation (in ‰) that occurs between each trophic level [39]. Δ was set at 2.8 ‰ [40]. δ^15^N_base_ was estimated using mean δ^15^N of the primary consumer fish species showing the lowest isotopic δ^15^N signal. δ^13^C_corr_ was calculated using the equation: δ^13^C_corr_ = δ^13^C_fish_ - δ^13^C_mpc_ ∕ CR_pc_. Where δ^13^C_fish_ is the carbon isotope signal of the focal fish species; δ^13^C_mpc_ is the mean primary consumer carbon isotope signal and CR_pc_ is the carbon range (δ^13^C_max_ - δ^13^C_min_) for all herbivore and detritivore individuals sampled. We used primary consumers as baselines because they integrate spatial and temporal variations in the isotopic signatures of primary producers [39]. δ^15^N_base_, δ^13^C_corr_, δ^13^C_mpc_, and CR_pc_ were estimated for each individual lake. Lake had no significant effect (ANOVA, p > 0.05) on mean TP and δ^13^C_corr_ when comparing colonized lakes (Mentiroso and Miraflores), thus, data from both colonized lakes were combined for posterior analyses. To estimate the relative contribution of detritivorous, herbivorous and invertivorous fishes to the dietary composition of piscivorous fishes in non-colonized and colonized environments, we fitted Bayesian isotope mixing models using the package “MixSIAR” [41]. Mean trophic discrimination factor (TDF) values were set to 1.3 (SD = 0.3) ‰ and 2.8 (SD = 0.4) ‰ for δ^13^C and δ^15^N, respectively [40]. Data are provided in S2 Table, data from the colonized Lake Miraflores were excluded from this analysis due to the lack of sample from all prey fish guilds.

We used two-way ANOVA to assess interaction effects of lake type (colonized / non-colonized) and trophic guilds on TP using each fish individual as replicates. We then tested for differences in TP between trophic guilds within each of the lake types using one-way ANOVAs followed by Tukey’s HSD test. Before each ANOVA test performed, we verified data for normality using Shapiro-Wilk test. We calculated isotopic niche widths using the “Stable Isotope Bayesian Ellipses” in the R package SIBER [36]. The program calculates metrics describing the data in a δ^13^C - δ^15^N space for each lake fish populations: i.e., the total amount of isotopic niche area occupied (total area; TA), the SEA and the sample size-corrected SEA (SEA_C_). To test whether the isotopic niches of species from colonized and non-colonized lakes differed, Bayesian inference was used to generate a distribution of covariance matrices (based on 10,000 posterior draws) that describe the observed data, and to calculate the posterior Bayesian estimates of the SEA (SEAb). We calculated the probability that SEAb in colonized environments is smaller or larger than SEAb in the non-colonized environment by comparing each pair of posterior draws and determining which is smaller or larger. The proportion of draws that are smaller (or larger) is a direct proxy for the probability of one group’s posterior distribution SEAb to be smaller (or larger) than the other [36,42,43]. All Data analyses were performed using R 4.2.2 [44].

## Results

A comparison of δ^13^C and δ^15^N values across the three lakes studied revealed that basal carbon sources tended to show lower δ^13^C values in Lake Miraflores, while Lake Tiuco showed higher δ^15^N values (S3 Table). Significant interaction effects between lakes and basal carbon sources were detected for both δ^13^C and δ^15^N isotopes (two-way ANOVA, p-values < 0.001) (S4 Table).

Primary consumers (i.e. herbivorous and detritivorous fish species) were at the bottom of the food chain with TPs varying from 2.0 to 2.6. The invertivorous fish *Triportheus albus* showed intermediate TP values from 2.6 to 2.7, and the piscivorous species showed the highest TPs varying from 2.9 to 3.6 (Table 1). Significant interaction effects between trophic guilds and types of lake were identified for TP (two-way ANOVA, p < 0.05), showing that the effect of trophic guilds on TP values varied between types of lakes (S5 Table). Subsequent analyses were performed independently for each type of lake. Significant differences among fish trophic guilds were observed within colonized lakes, with detritivores exhibiting the lowest trophic level, followed by herbivores, invertivores, and finally piscivores (p-values < 0.05). This pattern remained similar in the non-colonized lake except that detritivores and herbivores TPs were not significantly different (p = 0.8) (S6 Table). Pairwise comparisons of TP in colonized and non-colonized lakes for each trophic guild showed no significant differences (p-values > 0.05) except for piscivorous fishes, the species belonging to this guild exhibiting significantly higher TPs in non-colonized compared to colonized lakes (p < 0.001; Tables 1 and S5). For piscivores from the non-colonized Lake Tiuco, median posterior estimates from isotope mixing models indicated that invertivorous prey-fish comprised the largest proportion of the diet (49%), followed by detritivorous and herbivorous prey-fish that contributed approximately 15% and 28%, respectively. In the colonized Lake Mentiroso, the diet of piscivorous fishes shifted towards a predominant consumption of detritivorous prey-fish that represented between 80% and 100% of the diet (Table 2).

**Table 2:** Proportional contribution (median [Mdn], standard deviation [SD], and 95% credible intervals [CI]) of prey groups to the diet of piscivorous fishes in *A*. *gigas* colonized and non-colonized lakes in the Bolivian Amazon basin.

	Non-colonized		Colonized	
	n	Mdn (SD) [95% CI]	n	Mdn (SD) [95% CI]
Detritivores	18	0.15 (0.16) [0.00–0.54]	22	1.00 (0.07) [0.80–1.00]
Herbivores	40	0.28 (0.33) [0.00–1.00]	29	0.00 (0.04) [0.00–0 .08]
Invertivores	12	0.49 (0.28) [0.00–0.96]	9	0.00 (0.06) [0.00–0.16]

**Fig 2:**
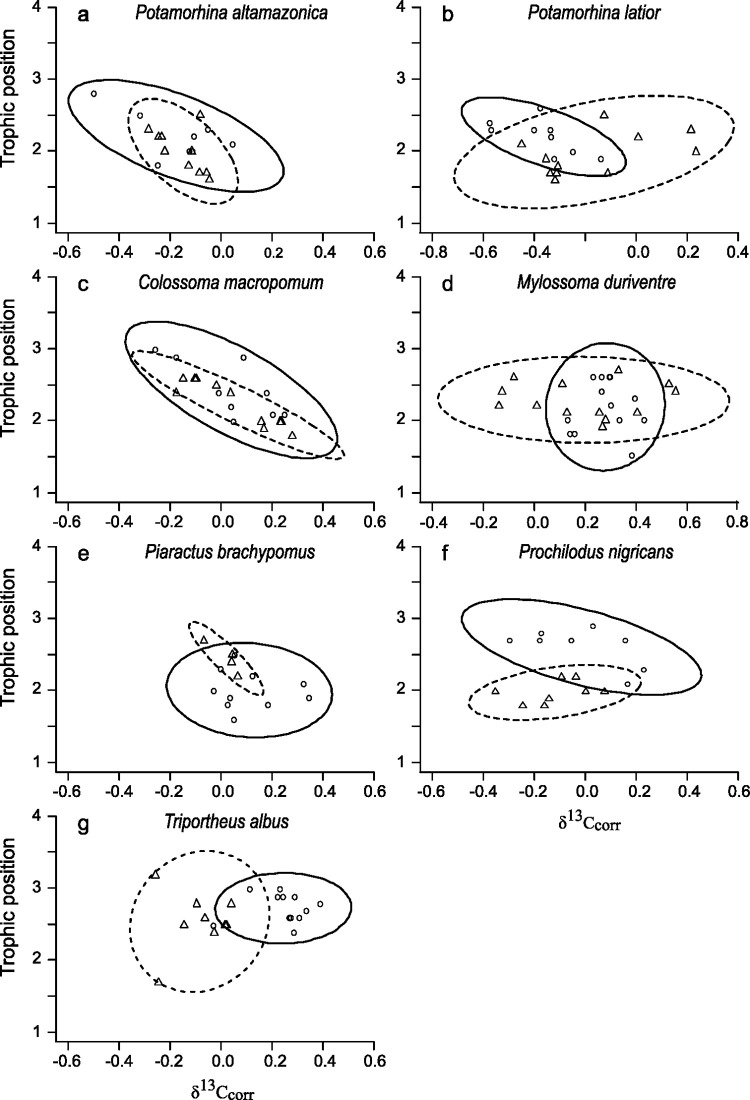
Isotopic niche of seven native fish species (detritivorous, herbivorous and invertivorous) in *Arapaima gigas* colonized and non-colonized lakes. Isotopic niche estimated from δ^13^C_corr_ and TP values for native detritivorous (a, b), herbivorous (c, d, e, f) and invertivorous (g) fish species. Each symbol (circles and triangles) represents an individual fish, while the ellipses denote the 95% credible interval. Circles and solid lines represent fish from the non-colonized lake (Mamoré River), and triangles and dashed lines represent fish from colonized lakes (Madre de Dios River).

**Fig 3:**
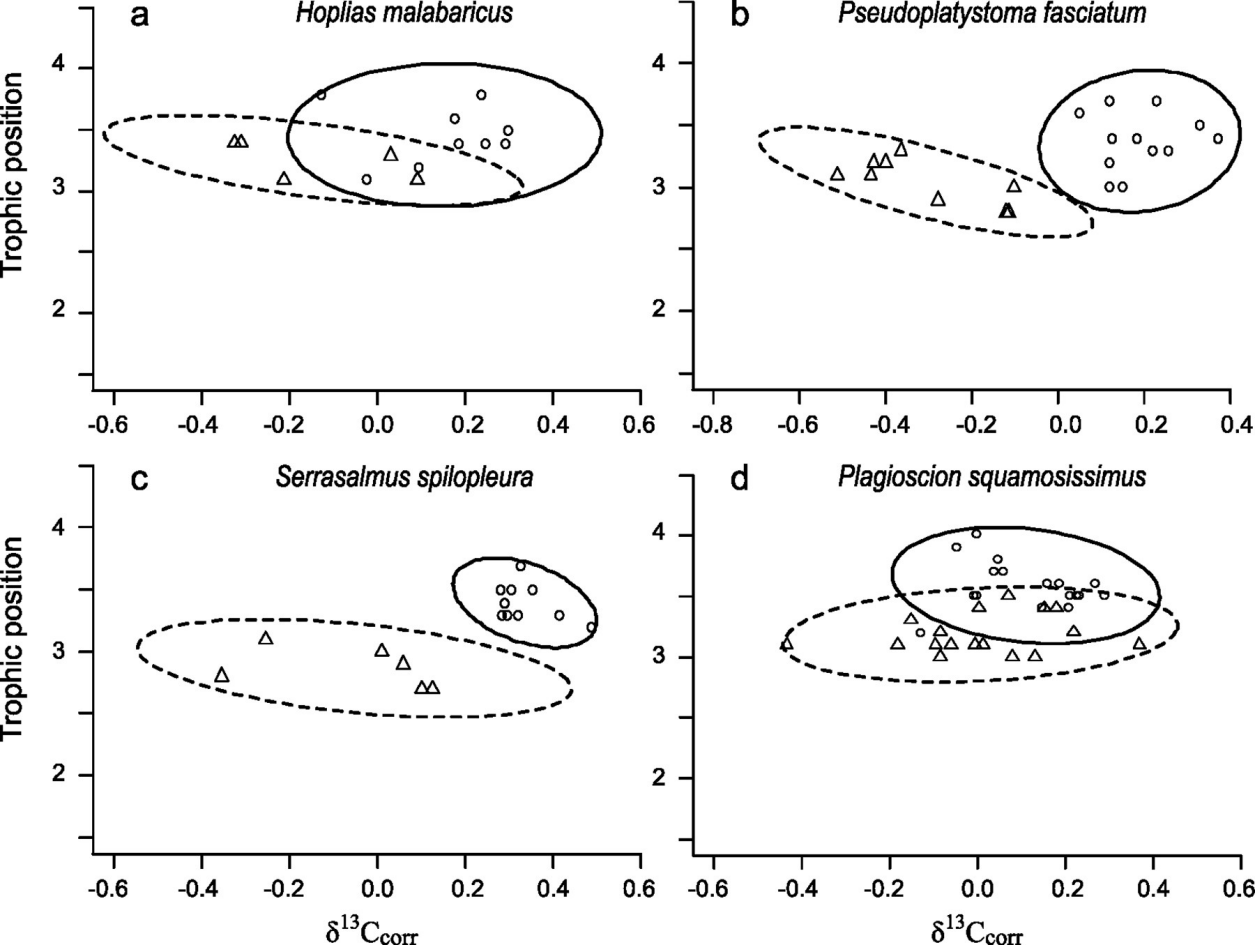
Isotopic niche of four native fish species (piscivorous) in *Arapaima gigas* colonized and non-colonized lakes. Isotopic niche estimated from δ^13^C_corr_ and TP values for native piscivorous fish species. Each symbol (circles and triangles) represents an individual fish, while the ellipses denote the 95% credible interval. Circles and solid lines represent fish from the non-colonized lake (Mamoré River), and triangles with dashed lines represent fish from colonized lakes (Madre de Dios River).

**Fig 4:**
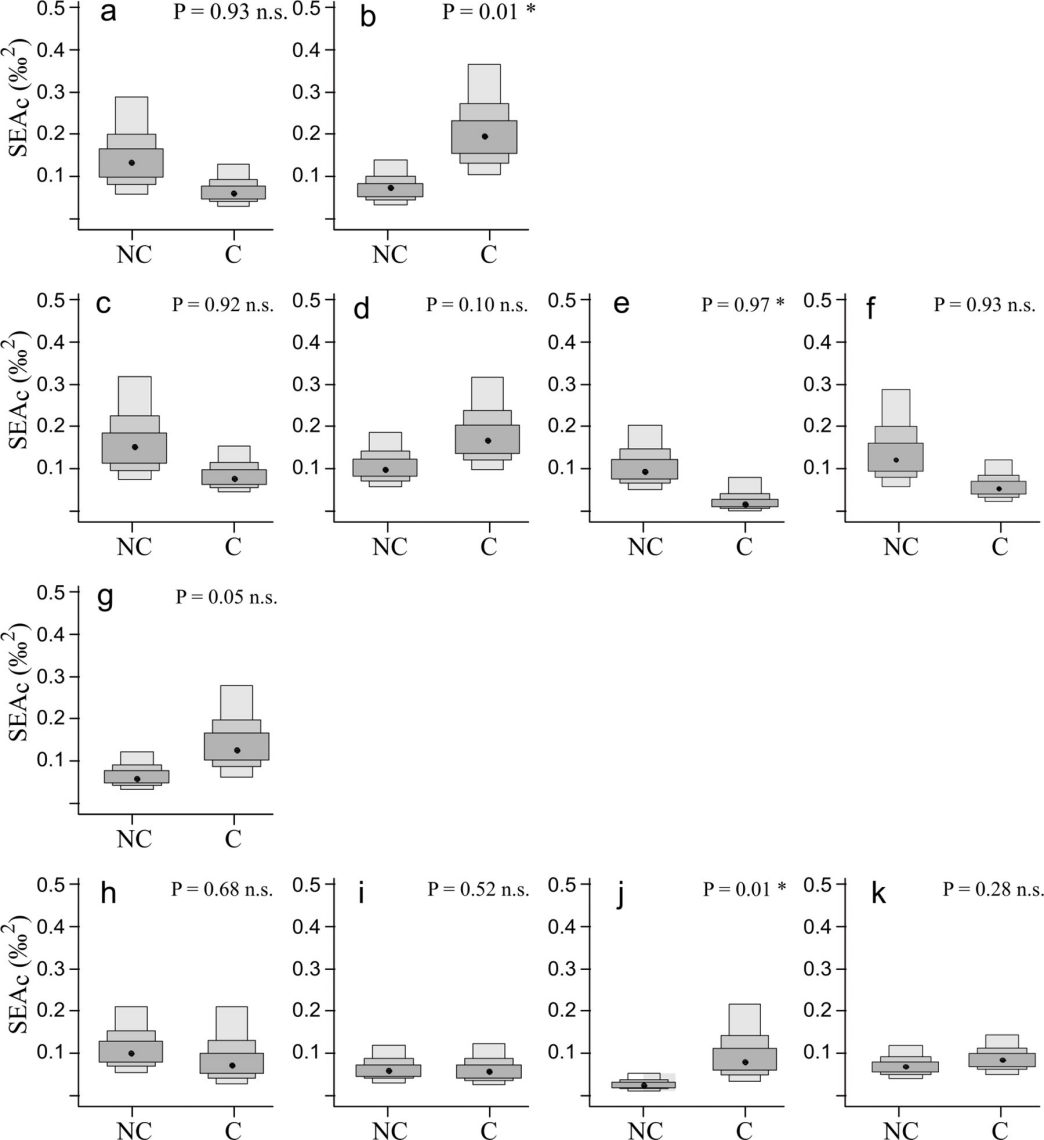
Estimated niche width for 11 native fish species in *Arapaima gigas* colonized and non-colonized lakes. Boxplots of the Bayesian posterior estimates of Bayesian Standard Ellipse Area (SEAb) native fish species in *A*. *gigas* colonized (C) and non-colonized (NC) lakes. Black dotes represent the mode, shaded boxes represent the 50%, 75% and 95% credible intervals from dark to light grey. P is the probability that SEAb in the colonized lakes is smaller than in the non-colonized one. A p-value ≤ 0.05 indicates a significantly smaller SEAb within the colonized lakes compared to the non-colonized one. P-values > 0.05 and < 0.95 indicate no significant difference in SEAb, while p-values ≥ 0.95 indicate a significantly smaller SEAb in the non-colonized site. Figures a (*P*. *altamazonica*) and b (*P*. *latior*) represent detritivorous species; figures c (*C*. *macropomum*), d (*M*. *duriventre*), e (*P*. *brachipomus*) and f (*P*. *nigricans*) represent herbivorous species; figure g (*T*. *albus*) represents invertivorous species; and figures h (*H*. *malabaricus*), i (*P*. *fasciatum*), j (*S*. *spilopleura*) and k (*P*. *squamosissimus*) represent piscivorous species.

SIBER analyses revealed that, irrespective of their trophic guild affiliation, the isotopic niche width of the majority of species did not exhibit significant differences between lakes colonized by *A*. *gigas* and the non-colonized lake (eight out of eleven species) (SEAb, 0.05 < p < 0.95). For two species, namely the detritivore *Potamorhina latior* and the piscivore *Serrasalmus spilopleura*, the isotopic niche width was significantly larger in the colonized environment (p-values < 0.05) and only one species, the herbivore *Piaractus brachipomus* (p = 0.97) showed a smaller isotopic niche width in the colonized environment compared to the non-colonized one (Table 3, Figs 2–4). However, concerning this last species, the smaller isotopic niche width noticed in the colonized environment compared to the non-colonized one is most probably the result of a niche width underestimation due to the small sample size (only 4 individuals sampled in the colonized lake), as SEA is strongly influenced by the number of individuals included in its calculation [45].

**Table 3:** Isotopic niche area (‰^2^) estimates for native fish species from non-colonized (River Mamoré) and colonized (Madre de Dios River) lakes.

	TA	SEA	SEAc	SEAb	95% CI
	Non-colonized lakes				
Detritivores					
*Potamorhina altamazonica*	0.21	0.13	0.16	0.14	0.06–0.29
*Potamorhina latior*	0.15	0.07	0.08	0.07	0.04–0.14
Herbivores					
*Colossoma macropomum*	0.25	0.15	0.17	0.14	0.08–0.31
*Mylossoma duriventre*	0.22	0.11	0.12	0.10	0.06–0.19
*Piaractus brachypomus*	0.20	0.11	0.12	0.10	0.06–0.21
*Prochilodus nigricans*	0.21	0.13	0.15	0.13	0.06–0.28
Invertivores					
*Triportheus albus*	0.16	0.07	0.08	0.07	0.04–0.12
Piscivores					
*Hoplias malabaricus*	0.20	0.11	0.13	0.10	0.05–0.21
*Pseudoplatystoma fasciatum*	0.14	0.07	0.08	0.06	0.04–012
*Serrasalmus spilopleura*	0.05	0.03	0.03	0.02	0.01–0.06
*Plagioscion squamosissimus*	0.18	0.07	0.08	0.07	0.04–0.12
	Colonized lakes				
Detritivores					
*Potamorhina altamazonica*	0.11	0.07	0.08	0.06	0.04–0.14
*Potamorhina latior*	0.38	0.20	0.22	0.19	0.11–0.35
Herbivores					
*Colossoma macropomum*	0.11	0.06	0.07	0.08	0.04–0.15
*Mylossoma duriventre*	0.37	0.18	0.20	0.17	0.10–0.31
*Piaractus brachypomus*	0.01	0.02	0.03	0.03	0.01–0.09
*Prochilodus nigricans*	0.10	0.06	0.07	0.06	0.03–0.13
Invertivores					
*Triportheus albus*	0.25	0.14	0.16	0.13	0.07–0.27
Piscivores					
*Hoplias malabaricus*	0.08	0.08	0.10	0.07	0.03–0.21
*Pseudoplatystoma fasciatum*	0.10	0.06	0.07	0.06	0.03–0.13
*Serrasalmus spilopleura*	0.11	0.09	0.12	0.08	0.04–0.22
*Plagioscion squamosissimus*	0.22	0.09	0.10	0.09	0.05–0.15

Estimates of isotopic niche area are given as total area (TA), standard ellipse area (SEA), sample size-corrected standard ellipse area (SEAc) and the mode of the Bayesian (posterior) standard ellipse area (SEAb) estimates. Upper and lower 95% credible intervals (CI) indicate the uncertainty in the SEAb estimates calculated from the posterior distributions of the fitted ellipses.

## Discussion

Here we investigated the potential influence of the non-native fish *Arapaima gigas* on TP and isotopic niche width of native fish species by comparing colonized (Madre de Dios River) and non-colonized lakes (Mamoré River). Our study reveals, regardless of the presence of *A*. *gigas*, a robust alignment between TP values and the trophic guilds determined *a priori* from literature, with primary consumers (detritivores and herbivores) showing the lowest values, invertivorous species intermediate values, and piscivorous species showing the highest values. However, significant lower TP values were noticed for piscivorous species inhabiting colonized lakes compared to the non-colonized lake. Our estimations of the relative contribution of the different fish guilds to the diet of piscivorous fish showed that this decreasing tendency in TP values was caused by a shift in the diet of piscivorous species toward prey-fish located lower in the food chain, with detritivorous fishes representing the largest proportion of their diet in the colonized Lake Mentiroso. These findings align with previous research suggesting that introduced species can sometimes force native species to feed on lower trophic levels [24,46].

Such alterations in trophic dynamics may stem from non-exclusive mechanisms such as interference competition (i.e. *A*. *gigas*, as a territorial and dominant species, may force other piscivores to use poorer foraging areas) and/or induced scarcity of prey resources (i.e. fewer prey-fish individuals available in colonized lakes). The first mechanism described above probably acts in our case as we also observed a shift towards lower δ^13^C_corr_ values for piscivorous species in colonized compared to the non-colonized lakes (see Fig 3), suggesting a displacement of these species to different and potentially sub-optimal feeding habitats. The fact that lower TPs were found for piscivorous species but not for species belonging to the remaining trophic guilds is consistent with *A*.* gigas* feeding habits (a generalist species with piscivorous tendencies [16,18,47]) as resources are supposed to be sufficiently abundant in the three lakes to reduce the likelihood of direct competition.

Conversely, the isotopic niche width did not differ significantly between species occurring in *A*. *gigas* colonized and non-colonized environments. These findings contrast with our original hypothesis predicting a reduction in the isotopic niche width of native species due to potential competition and resource constraints imposed by *A*. *gigas* in the colonized lakes [24,26]. Even if the absence of a significant reduction in isotopic niche widths despite high niche overlaps between native fish species and *A*. *gigas* [16] cannot formally be interpreted as an absence of competitive interaction between *A*. *gigas* and native fish assemblages, this absence of niche width reduction allows at least to conclude that trophic niche space of native fishes is resistant to change from colonization by *A*. *gigas*. Due to the historical presence and diversity of piscivores in the native fish assemblages [32,48,49], prey-fish may have developed effective antipredator strategies prior to colonization, potentially reducing the impact of this new predator [50,51].

While we acknowledge that our study suffers limitation concerning sample size (i.e. number of lakes and species analyzed, number of individuals sampled) and that the isotopic niche needs to be interpreted with caution as it does not fully reflect the true trophic niche of species [21], our results based on species TP and isotopic niche width suggest that the ecological impact of *A*. *gigas* on native fish in the two colonized lakes is rather weak for most native species. However, there is a notable exception for piscivores, which exhibit a significant decline in TP following *A*. *gigas* establishment. Further analyses on potential indirect effects, such as changes in community and/or food web structure following *A*. *gigas* colonization may provide valuable insights into other potential ecological impacts that this introduced species may generate in Bolivian waters.

## Supporting information

S1 Table Number of individuals (n), standard length (SL) and standard deviation (SD) of fish captured in lakes colonized and non-colonized by *Arapaima gigas*, located in the floodplains of rivers Mamore and Madre de Dios, respectively. (DOCX)

S2 Table Number of individuals (n), mean, median (Mdn), standard deviation (SD) and confidence intervals (CI) for fish trophic guilds sampled in lakes colonized and non-colonized by *Arapaima gigas*, located in the floodplains of rivers Mamore and Madre de Dios, respectively. (DOCX)

S3 Table Sample size (n) and mean ẟ^13^C and ẟ^15^N values (± SD) for the carbon sources from non-colonized (Lake Tiuco) and *Arapaima gigas* colonized lakes (Lakes Mentiroso and Miraflores), located in the floodplains of rivers Mamore and Madre de Dios, respectively. (DOCX)

S4 Table Results of two-way ANOVA, testing the effect of basal carbon source (C4-macrophytes, C3-macrophytes, POM, and terrestrial vegetation) and lake on ẟ^13^C and ẟ^15^N values. Significant differences for: *P<0.05; **P<0.01; ***P<0.001.
(DOCX)

S5 Table Results of two-way ANOVA, testing the effect of ‘trophic guild’ (detritivores, herbivores, invertivores and piscivores) and ‘Type of lake’ (colonized and non-colonized by *Arapaima gigas*) on trophic position (TP); and post hoc Tukey’s HSD tests pairwise comparisons of trophic guilds in colonized and non-colonized lakes. Significant differences for: *P<0.05; **P<0.01; ***P<0.001; NS: no significant differences for P>0.05.
(DOCX)

S6 Table Results of one-way ANOVAs and post hoc Tukey’s HSD test, testing for the effect of “trophic guild’ on TP in lakes colonized and non-colonized by *Arapaima gigas*. Significant differences for: *P<0.05; **P<0.01; ***P<0.001; N.S.: no significant differences for P>0.05.
(DOCX)
