# Supplementary material for: Effects of the non-native Arapaima gigas on native fish species in Amazonian oxbow lakes (Bolivia)
Source: PLoS One. 2025 Jan 2;20(1):e0314359. doi: 10.1371/journal.pone.0314359 (PMC11695033; doi:10.1371/journal.pone.0314359)
Supplement: S1 Table — (DOCX) [file pone.0314359.s001.docx]

**S1 Table.** Number of individuals (n), standard length (SL) and standard deviation (SD) of fish captured in lakes colonized and non-colonized by *Arapaima gigas*, located in the floodplains of rivers Mamore and Madre de Dios, respectively.

|  | L. Tiuco (Non -colonized) | | | L. Mentiroso (Colonized) | | | L. Miraflores (Colonized) | | |
| --- | --- | --- | --- | --- | --- | --- | --- | --- | --- |
| Species | n | mean SL (mm) | SD SL | n | mean SL (mm) | SD SL | n | mean SL (mm) | SD SL |
| *Arapaima gigas* |  |  |  | 32 | 1306 | 491 | 7 | 975 | 313 |
| *Potamorhina altamazonica* | 8 | 138 | 40 | 10 | 210 | 12 |  |  |  |
| *Potamorhina latior* | 10 | 137 | 10 | 12 | 199 | 10 |  |  |  |
| *Colossoma macropomum* | 9 | 456 | 101 | 12 | 384 | 122 |  |  |  |
| *Mylossoma duriventre* | 13 | 169 | 37 | 13 | 131 | 25 |  |  |  |
| *Piaractus brachypomus* | 10 | 349 | 57 | 4 | 352 | 75 |  |  |  |
| *Prochilodus nigricans* | 8 | 308 | 44 |  |  |  | 8 | 339 | 44 |
| *Triportheus albus* | 12 | 145 | 18 | 9 | 234 | 7 |  |  |  |
| *Hoplias malabaricus* | 9 | 362 | 60 | 5 | 334 | 141 |  |  |  |
| *Pseudoplatystoma fasciatum* | 12 | 666 | 191 | 9 | 628 | 182 |  |  |  |
| *Serrasalmus spilopleura* | 10 | 141 | 12 | 6 | 129 | 14 |  |  |  |
| *Plagioscion squamosissimus* | 17 | 317 | 78 |  |  |  | 17 | 425 | 78 |
