# Supplementary material for: Effects of the non-native Arapaima gigas on native fish species in Amazonian oxbow lakes (Bolivia)
Source: PLoS One. 2025 Jan 2;20(1):e0314359. doi: 10.1371/journal.pone.0314359 (PMC11695033; doi:10.1371/journal.pone.0314359)
Supplement: S2 Table — (DOCX) [file pone.0314359.s002.docx]

**S2 Table.** Number of individuals (n), mean, median (Mdn), standard deviation (SD) and confidence intervals (CI) for fish trophic guilds sampled in lakes colonized and non-colonized by *Arapaima gigas*, located in the floodplains of rivers Mamore and Madre de Dios, respectively.

|  |  | δ^13^C |  |  |  | δ^15^N |  |  |  |
| --- | --- | --- | --- | --- | --- | --- | --- | --- | --- |
| Fish guild | n | Mean | Mdn | SD | 95 % CI | Mean | Mdn | SD | 95 % CI |
| Non-colonized | | | | | | | | | |
| Detritivores | 18 | -35.39 | -35.93 | 2.34 | -39.28 ̶ -32.62 | 6.98 | 7.05 | 0.73 | 5.88 ̶ 8.30 |
| Herbivores | 40 | -29.95 | -29.67 | 2.42 | -35.04 ̶ -26.37 | 7.17 | 7.03 | 1.08 | 5.23 ̶ 8.79 |
| Invertivores | 12 | -28.32 | -27.97 | 1.46 | -31.46 ̶ -26.58 | 8.42 | 8.41 | 0.60 | 7.47 ̶ 9.24 |
| Piscivores | 48 | -29.13 | -28.87 | 1.85 | -33.15 ̶ -26.16 | 10.49 | 10.44 | 0.61 | 9.25 ̶ 11.69 |
| Colonized | | | | | | | | | |
| Detritivores | 22 | -31.90 | -31.64 | 1.42 | -33.88 ̶ -28.74 | 6.04 | 5.97 | 0.85 | 4.95 ̶ 7.44 |
| Herbivores | 29 | -29.60 | -29.71 | 1.65 | -31.83 ̶ -26.17 | 6.81 | 7.08 | 0.76 | 5.67 ̶ 7.87 |
| Invertivores | 9 | -31.26 | -31.08 | 0.93 | -32.37 ̶ -30.26 | 7.59 | 7.56 | 1.12 | 5.57 ̶ 9.16 |
| Piscivores | 20 | -32.12 | -32.52 | 1.68 | -34.45 ̶ -29.65 | 8.96 | 9.01 | 0.61 | 8.07 ̶ 9.95 |
