# Supplementary material for: Effects of the non-native Arapaima gigas on native fish species in Amazonian oxbow lakes (Bolivia)
Source: PLoS One. 2025 Jan 2;20(1):e0314359. doi: 10.1371/journal.pone.0314359 (PMC11695033; doi:10.1371/journal.pone.0314359)
Supplement: S3 Table — (DOCX) [file pone.0314359.s003.docx]

**S3 Table**. Sample size (n) and mean ẟ^13^C and ẟ^15^N values (± SD) for the carbon sources from non-colonized (Lake Tiuco) and *Arapaima gigas* colonized lakes (Lakes Mentiroso and Miraflores), located in the floodplains of rivers Mamore and Madre de Dios, respectively.

| Carbon source | n | Mean ẟ^13^C ±SD |  | Mean ẟ^15^N ±SD |
| --- | --- | --- | --- | --- |
| Non-colonized lake | | | | |
| Lake Tiuco |  |  |  |  |
| C4-macrophytes | 4 | -12.9 ± 0.2 |  | 3.5 ± 1.5 |
| C3-macrophytes | 14 | -29.9 ± 0.8 |  | 4.6 ± 0.6 |
| POM | 6 | -32.6 ±1.0 |  | 5.5 ± 0.6 |
| Terrestrial vegetation | 4 | -30.0 ± 1.6 |  | 3.2 ± 2.2 |
| Colonized lakes | | | | |
| Lake Mentiroso |  |  |  |  |
| C4-macrophytes | 2 | -11.9 ± 0.2 |  | 1.7 ± 0.2 |
| C3-macrophytes | 4 | -29.1 ± 0. 9 |  | -0.7± 1.3 |
| POM | 3 | -32.3 ± 0.1 |  | 0.6 ± 0.0 |
| Terrestrial vegetation | 4 | -33.3 ± 2.0 |  | 3.0 ± 2.5 |
| Lake Miraflores |  |  |  |  |
| C4-macrophytes | 3 | -14.1 ± 0.1 |  | 4.7 ± 0.3 |
| POM | 4 | -35.7 ± 0.1 |  | 3.3 ± 0.2 |
| Terrestrial vegetation | 6 | -31.2 ± 1.7 |  | 1.3 ± 1.9 |
