# Supplementary material for: Effects of the non-native Arapaima gigas on native fish species in Amazonian oxbow lakes (Bolivia)
Source: PLoS One. 2025 Jan 2;20(1):e0314359. doi: 10.1371/journal.pone.0314359 (PMC11695033; doi:10.1371/journal.pone.0314359)
Supplement: S4 Table — Significant differences for: *P<0.05; **P<0.01; ***P<0.001. (DOCX) [file pone.0314359.s004.docx]

**S4 Table**. Results of two-way ANOVA, testing the effect of basal carbon source (C4-macrophytes, C3-macrophytes, POM, and terrestrial vegetation) and lake on ẟ^13^C and ẟ^15^N values. Significant differences for: *P<0.05; **P<0.01; ***P<0.001.

|  | Source | *df* | *SS* | *MS* | *F* | *P* |
| --- | --- | --- | --- | --- | --- | --- |
| δ^13^C | Carbon source | 3 | 2602 | 868 | 744 | *** |
|  | Lake | 2 | 14 | 7 | 6 | ** |
|  | CS x L | 5 | 44 | 9 | 8 | *** |
|  | Residuals | 43 | 50 | 1 |  |  |
| δ^15^N | Carbon source | 16 | 5. | 3 |  | * |
|  | Lake | 97 | 48 | 30 |  | *** |
|  | CS x L | 62 | 12 | 8 |  | *** |
|  | Residuals | 70 | 2 |  |  |  |
