# Supplementary material for: Effects of the non-native Arapaima gigas on native fish species in Amazonian oxbow lakes (Bolivia)
Source: PLoS One. 2025 Jan 2;20(1):e0314359. doi: 10.1371/journal.pone.0314359 (PMC11695033; doi:10.1371/journal.pone.0314359)
Supplement: S5 Table — Significant differences for: *P<0.05; **P<0.01; ***P<0.001; NS: no significant differences for P>0.05. (DOCX) [file pone.0314359.s005.docx]

**S5 Table.** Results of two-way ANOVA, testing the effect of ‘trophic guild’ (detritivores, herbivores, invertivores and piscivores) and ‘Type of lake’ (colonized and non-colonized by *Arapaima gigas*) on trophic position (TP); and post hoc Tukey’s HSD tests pairwise comparisons of trophic guilds in colonized and non-colonized lakes. Significant differences for: *P<0.05; **P<0.01; ***P<0.001; NS: no significant differences for P>0.05.

| Source | *df* | *SS* | *MS* | *F* | *P* |
| --- | --- | --- | --- | --- | --- |
| Guild | 3 | 61.6 | 20.6 | 255.3 | *** |
| Type of lake | 1 | 2.5 | 2.5 | 31.6 | *** |
| G x T | 3 | 0.9 | 3.7 | 3.7 | * |
| Residuals | 215 | 17.3 |  |  |  |

| Tukey’s HSD test |  |
| --- | --- |
| Source | *P* |
| Detritivore (colonized) – Detritivore (non-colonized) | N.S. |
| Herbivore (colonized) – Herbivore (non-colonized) | N.S. |
| Invertivore (colonized) – Invertivore (non-colonized) | N.S. |
| Piscivore (colonized) – Piscivore (non-colonized) | *** |
