# Supplementary material for: Effects of the non-native Arapaima gigas on native fish species in Amazonian oxbow lakes (Bolivia)
Source: PLoS One. 2025 Jan 2;20(1):e0314359. doi: 10.1371/journal.pone.0314359 (PMC11695033; doi:10.1371/journal.pone.0314359)
Supplement: S6 Table — Significant differences for: *P<0.05; **P<0.01; ***P<0.001; N.S.: no significant differences for P>0.05. (DOCX) [file pone.0314359.s006.docx]

**S6 Table.** Results of one-way ANOVAs and post hoc Tukey’s HSD test, testing for the effect of “trophic guild’ on TP in lakes colonized and non-colonized by *Arapaima gigas*. Significant differences for: *P<0.05; **P<0.01; ***P<0.001; N.S.: no significant differences for P>0.05.

|  | Source | *df* | *SS* | *MS* | *F* | *P* |
| --- | --- | --- | --- | --- | --- | --- |
| Colonized | Guild | 3 | 22.0 | 7.3 | 99.1 | *** |
|  | Residuals | 101 | 7.5 | 0.01 |  |  |
| Non-colonized | Guild | 3 | 38.5 | 12.8 | 149 | *** |
|  | Residuals | 114 | 9.8 | 0.1 |  |  |

| Tukey’s HSD test |  |  |
| --- | --- | --- |
| Source | Non-colonized | Colonized |
| Detritivore - Herbivore | N.S. | * |
| Detritivore - Invertivore | *** | *** |
| Detritivore - Piscivore | *** | *** |
| Invertivore - Herbivore | *** | ** |
| Herbivore - Piscivore | *** | *** |
| Invertivore - Piscivore | *** | *** |
